# Regulation of Signaling Pathways Involved in the Anti-proliferative and Apoptosis-inducing Effects of M22 against Non-small Cell Lung Adenocarcinoma A549 Cells

**DOI:** 10.1038/s41598-018-19368-0

**Published:** 2018-01-17

**Authors:** Yao Yuan, Jiewei Wu, Bailin Li, Jia Niu, Haibo Tan, Shengxiang Qiu

**Affiliations:** 1 Key Laboratory of Plant Resources Conservation and Sustainable Utilization, Guangdong Provincial Key Laboratory of Applied Botany, South China Botanical Garden, Chinese Academy of Sciences, Guangzhou, 510650 P. R. China; 2 University of Chinese Academy of Sciences, Beijing, 100049 P. R. China

## Abstract

The compound 29-(4-methylpiperazine)-luepol (M22), a novel derivative of lupeol has shown anti-proliferative effects against the human non-small cell lung cancer A549 cell line. M22 showed significant anti-proliferative activity at 6.80 μM and increased accumulation of G1 cells and effectively suppressed expression of the G1 arrest-related genes cyclins D1 and E1, CDK2 and CDC25A. This was further confirmed by Western blotting demonstrating decreased cyclin D1 and CDC25A protein levels. Furthermore, M22 caused induction of apoptosis that downregulated the anti-apoptotic *BCL-*2 gene and increased expression of *BAX*, *CASP*3 and *CASP*9 as well as the *APAF*1 gene. The effect of caspase-induced apoptosis was confirmed by an increase in reactive oxygen species (ROS), loss of mitochondrial membrane potential (MMP). Taken together, our findings indicated that M22 possessed potent anti-proliferative and apoptotic activities.

## Introduction

Lung cancer is one of the most prevalent malignant diseases and has a low 5-year survival rate compared to other common types of cancer including prostate (99%), breast (89%) and colorectal (65%). Non-small cell lung cancer has been the leading cause of all lung cancer deaths since the 1980s^1^. China’s increasing industrialization and air pollution will result in a dramatic increase in lung cancer patients in the next few years. Traditional treatments with surgery, chemotherapy, radiotherapy and targeted EGFR or HER-2^2^ therapies may not be sufficient to address these changes.

**Figure 1:**
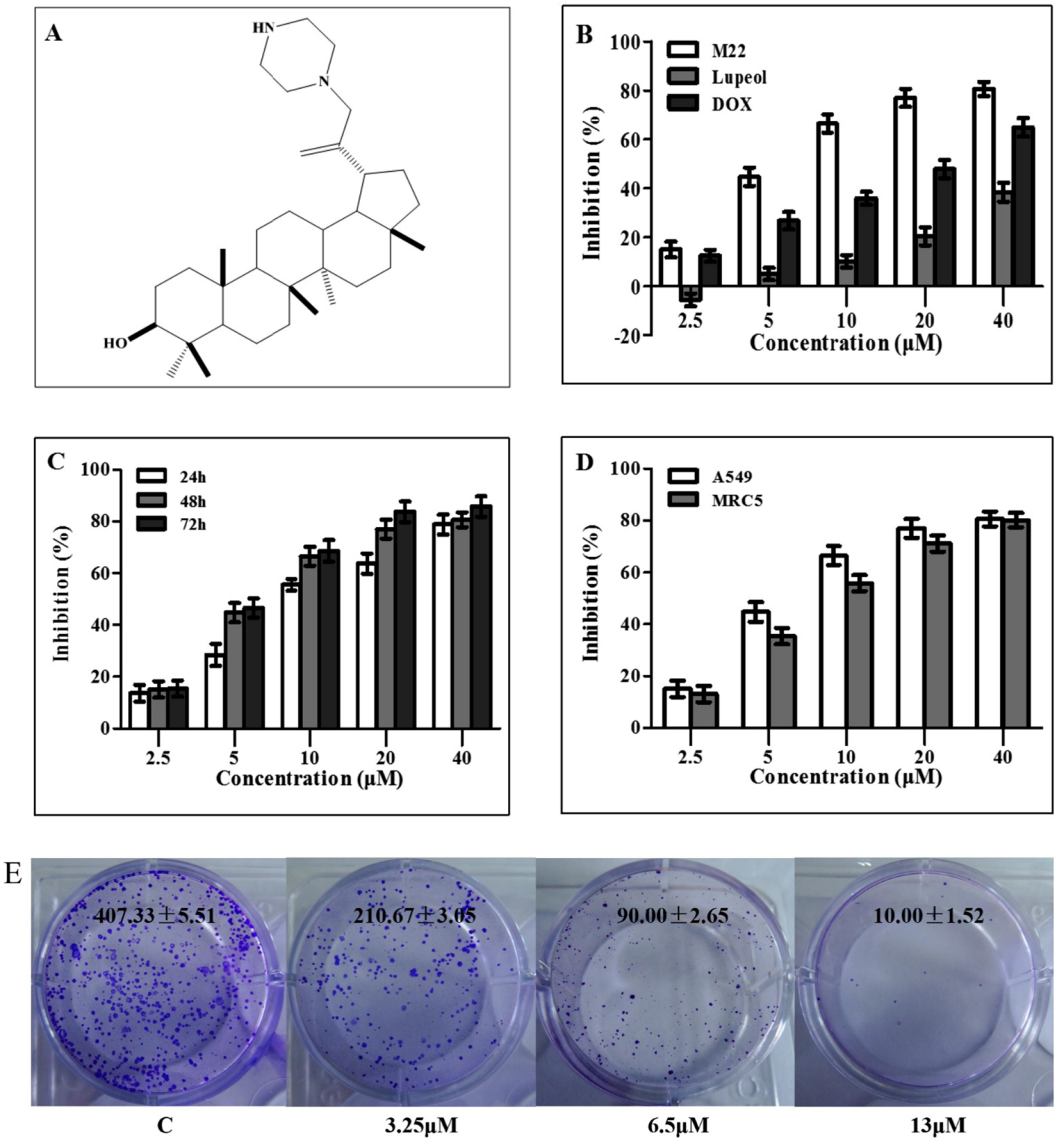
Chemical structure and cytotoxicity of M22. (**A**) Chemical structure of compound M22. (**B**–**C**) Dose- and time-dependent effect of M22 on inhibition of A549 cells. Cell viability was analyzed by MTT assay. (**D**) A549 and MRC5 cells were cultured with various concentrations of M22 for 48 h and cell inhibition was analyzed by MTT assay. The experiment was repeated three times and the data are presented as mean ± S.D. (**E**) Colony forming ability of A549 cells was inhibited by M22 (3.25 μM, 6.5 μM and 13 μM) treatment for 10 days.

The compound 29-(4-methylpiperazine)-luepol (M22) is a derivative of Lup-20(29)-en-3ß-ol (Lupeol) isolated from *Pinus luchuensis* stems and other medicinal plants and fruit^3,4^ (Fig. 1A). It was reported to have cytotoxic, anti-oxidant, anti-inflammatory as well as anti-tumor effects^5,6^. M22 could affect numerous cellular signaling processes such as apoptosis induction and growth inhibition of tumor cells. These effects included activation of NF-kappa B signaling as well as the protein kinases C (PKCα) and B (Akt), ornithine decarboxylase (ODC), phosphatidylinositol-3-kinase (PI3K) as well as mitogen activated protein kinases (MAPKs)^7–9^. It could also inhibit the anticancer chemotherapy target DNA topoisomerase II, but its specific activity was low thus limiting its application^10,11^. We therefore modified the structure of lupeol to enhance its activity.

Earlier we reported that a new derivative of lupeol-3β-O-succinyl-lupeol (LD9-4) induced autophagy through the mTOR signaling pathway in the human non-small lung cancer cell lines (A549)^12^. In the present study, potential anti-cancer activities of a new derivative (M22) were evaluated and its anti-proliferative, apoptotic properties and mechanism of action were also accessed.

## Results

### Cytotoxic potential of M22

Effect of M22 on four cancer cell lines, A549 (NSCL), SW480 (human gastric carcinoma cell line), HepG2 (human hepatocellular carcinoma cell line) and HeLa (human cervix carcinoma cell line) were studied using MTT assay (Table 1). Exposure of A549 cells to M22 (0–40 μM) resulted in a dose dependent inhibition of cell proliferation up to 80% (Fig. 1B) over 48 h with an IC_50_ value of 6.80 μM, which was significantly lower than that of parent compound - lupeol (35.69 μM) and the positive control medicine - DOX (25.43 μM). The results revealed that M22 inhibited A549 cell proliferation in a time- and dose-dependent manner (Fig. 1C). M22 was also added to human normal embryonic lung fibroblast cells (MRC5). The results showed that M22 was almost equal toxicity to both A549 and MRC5 cells. However, at concentrations of 5 μM or 10 μM, M22 was slightly more cytotoxic in A549 cell lines than that in MRC5 cell lines (Fig. 1D).

**Table 1:** IC_50_ values of lupeol derivatives M22 against four human cancer cell lines for 48 h.

Sample	IC_50_ (μM)			
	A549	SW480	HepG2	HeLa
M22	6.80 ± 0.33	9.74 ± 0.43	9.64 ± 0.25	7.18 ± 0.36
Lupeol	35.69 ± 0.75	48.51 ± 0.82	45.23 ± 0.67	34.76 ± 0. 64
Doxorubicin	25.43 ± 0.57	>50	>50	25.23 ± 0. 53

We further tested the long-term effect of M22 to inhibit cell viability in A549 cells using colony formation assay. The results revealed that the potent inhibitory activity of M22 in concentration-dependent manner. (Fig. 1E).

### Effect of M22 on cell cycle progression

**Figure 2:**
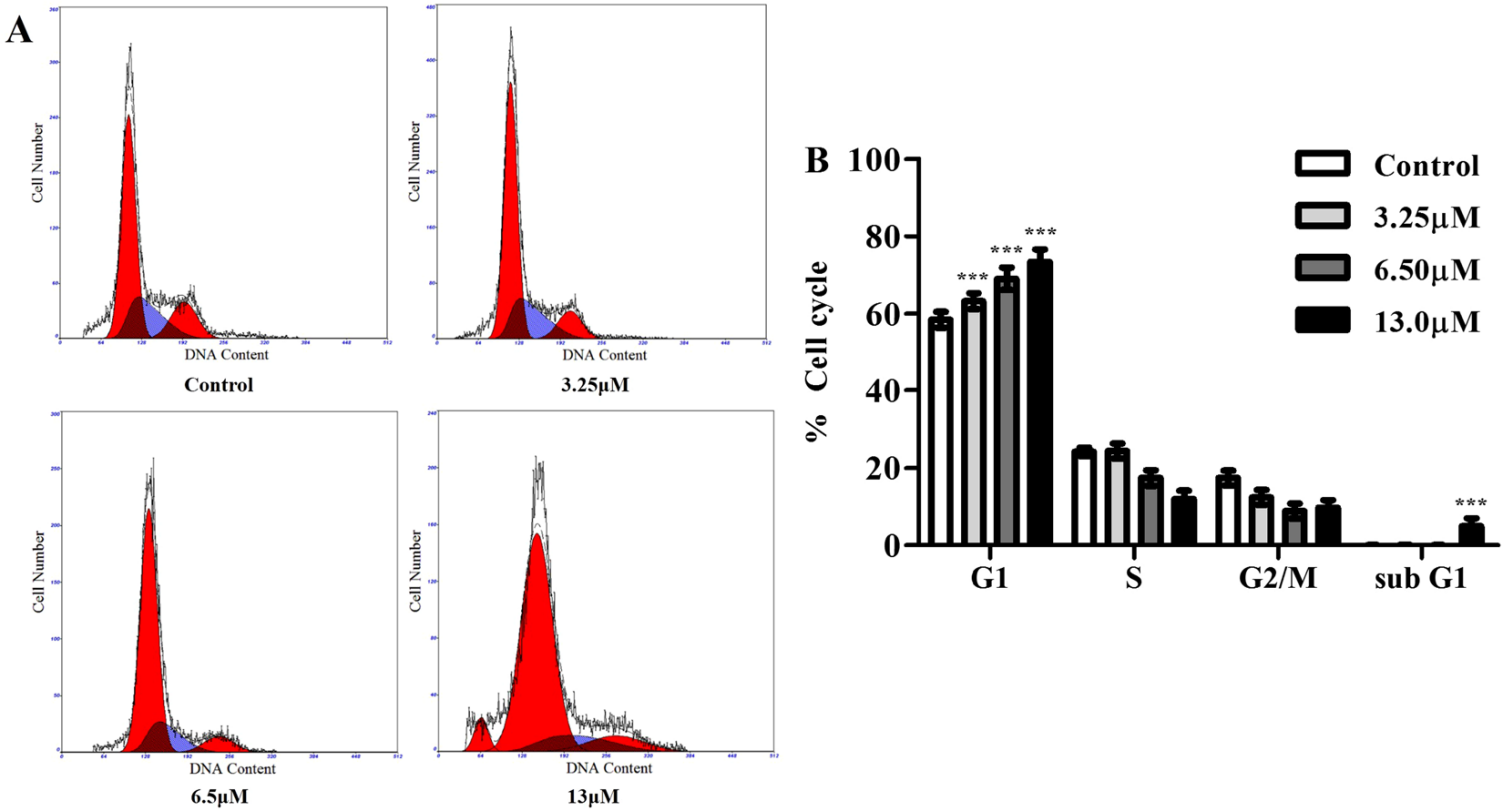
Flow cytometry analysis histograms of G0/G1-phase arrest in 24 h M22 treated A549 cells. (**A**) The G0/G1 peak increases with corresponding decrease in S and G2 peak in dose dependent manner. M22 treated cells at 13 μM showing an increased sub G1 peak indicating apoptosis. (**B**) The percent of cells in different phases of cell cycle are represented by bar diagram. Data are from 3 independent experiments and analyzed by one-way analysis of variance, ***p < 0.001 compared to control. PI fluorescence was measured using a flow cytometer with FL-2 filter.

To determine the effect of M22 on cell cycle arrest and cell death in lung cancer cells, A549 cells were treated with various concentrations of M22 for 24 h. The cells were analyzed by flow cytometry and stained with propidium iodide (PI). As shown in Fig. 2, M22 addition also resulted in a G1/G1S block reaching almost 75%. In addition, the sub G1 population was increased to 4.94% at 13 μM M22 at 24 h, indicating the drug might induce apoptosis in A549 cells (Fig. 2).

### Effect of M22 on genes and proteins involved in G1 to S transition

**Figure 3:**
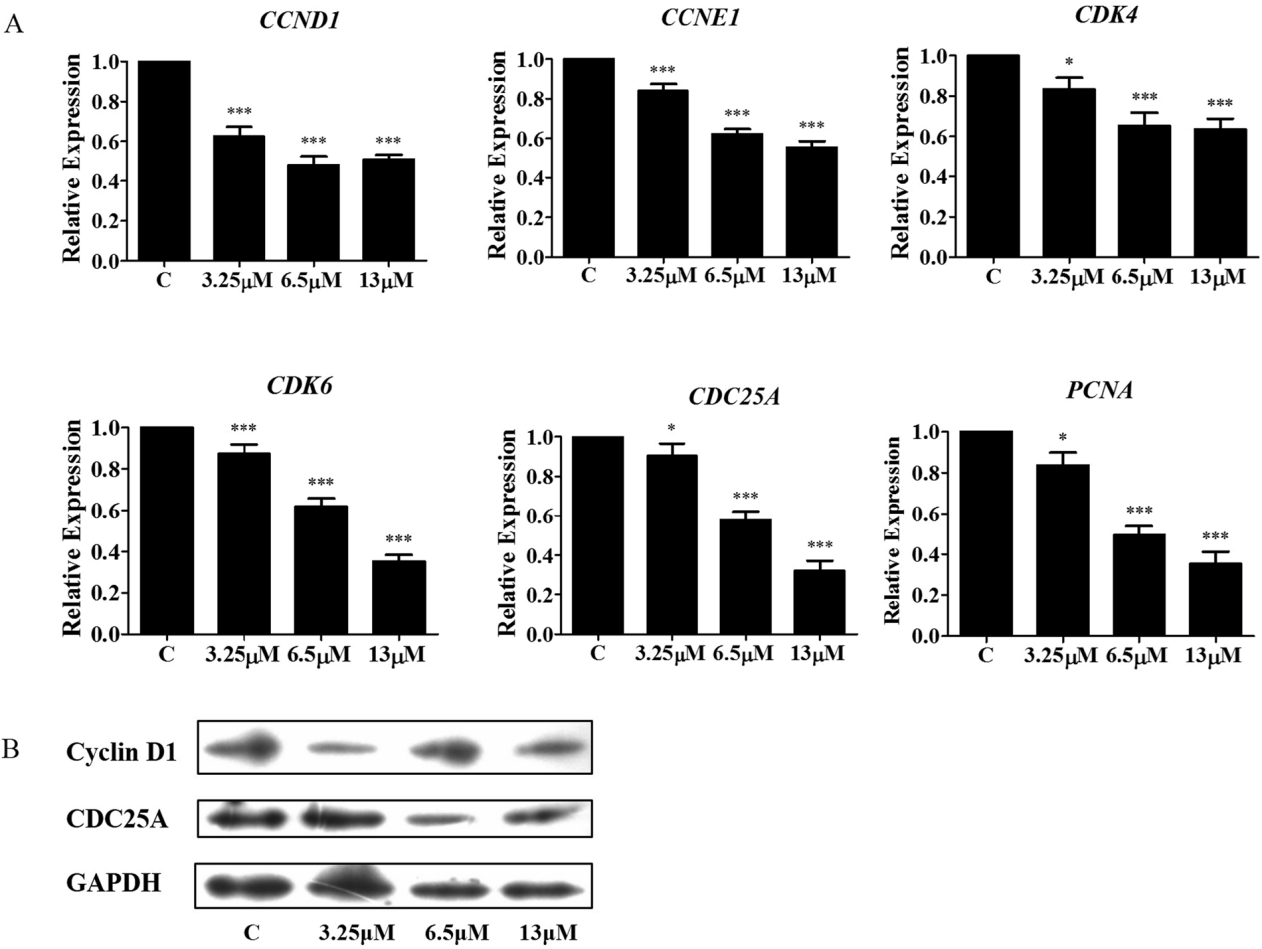
M22 regulates G0/G1 arrest through a negative feedback mechanism. (**A**) M22 attenuates mRNA expression of Cyclin D1, Cyclin E1, CDK4, CDK6, CDC25A and PCNA genes in A549 cells for 24 h. RNA was isolated and analsis was performed to detect by qRT-PCR. *Means P < 0.01, ***means P < 0.0001. (**B**) M22 down-regulates the expression of Cyclin D1 and CDC25C protein in A549 cells by Western blotting.

Consistently, M22 treatment significantly decreased *CCND1* and *CDK4* expression (Fig. 3). This was consistent with the role of cyclin D1 to bind and activate cyclin-dependent kinases 4 and 6 (CDK4 and CDK6) which regulated the G1/S transition^13^.

In conjunction with these findings, mRNA levels of the genes for G1-related cyclin E1, PCNA and CDC25A were dramatically decreased by M22 treatment compared to the untreated control. M22 also induced down regulation of the CDC25A and cyclin D1 proteins (Fig. 3).

### M22 induces apoptosis in A549 cells

**Figure 4:**
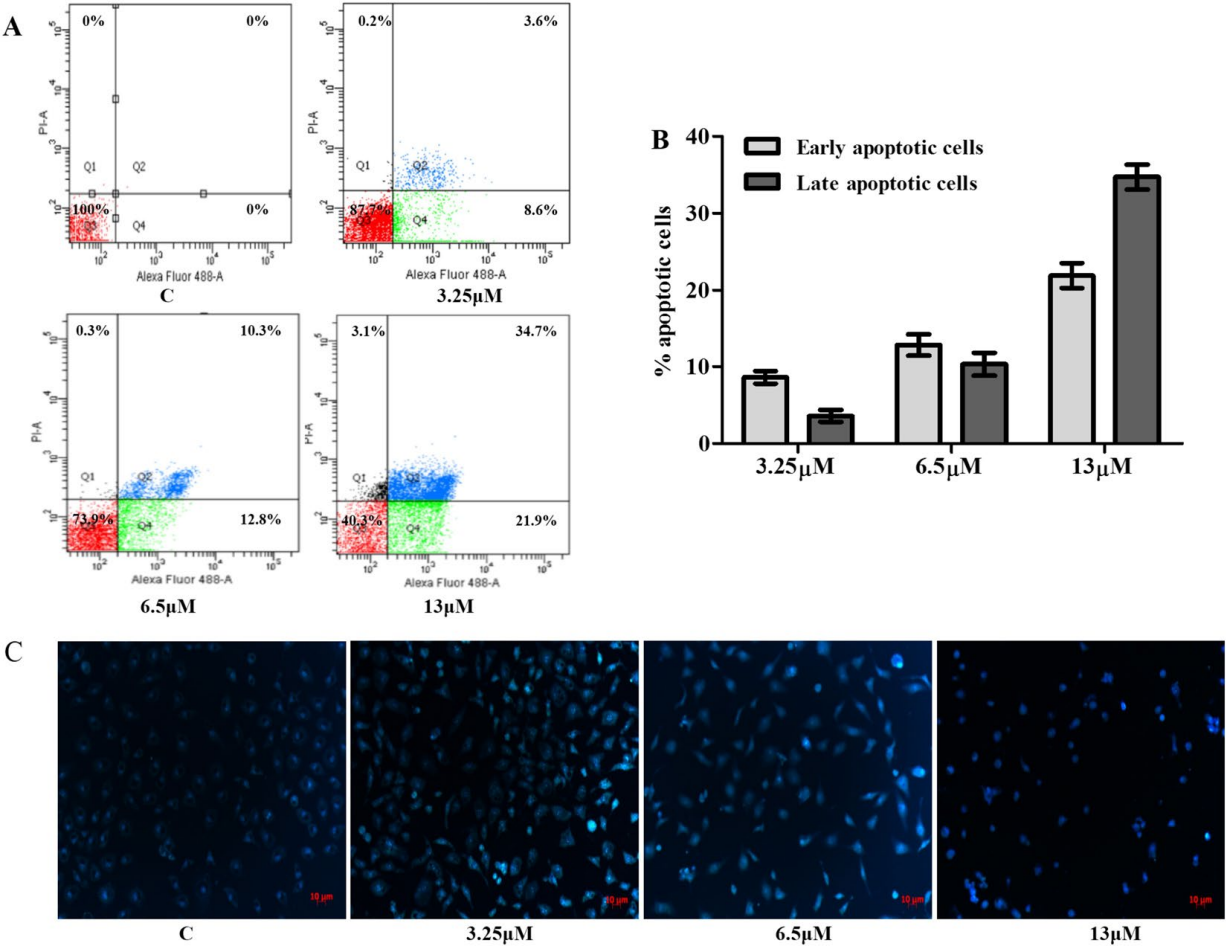
Detection of apoptosis induced by M22 by flow cytometry and confocal microscopy. (**A**) Evaluation of apoptosis by Annexin V/PI dual staining assay. Numbers of Annexin V-and PI-positive cells were determined using flow cytometry. Quadrant 1: necrotic cells; Quadrant 2: late-apoptotic cells; Quadrant 3: viable cells; Quadrant 4: early apoptotic cells. (**B**) The percent of apoptotic cells are represented by bar diagram. (**C**) Morphologic changes of A549 cells treated by M22 for 48 h were fixed and stained with Hoechst 33258. M22 treated cells show apoptotic, condensed and fragmented fluorescent nuclei. Bars, 10 μm.

We also found a significant increase of early apoptosis and a progressive increase of late apoptosis with increasing concentrations of M22 at 48 h. Apoptotic cells increased from 23% to 57% following M22 treatment in increasing dosages (Fig. 4A, Quadrant 2 and 4). Hoechst 33258 staining result recorded that most cells showed typical apoptosis characters in M22 treated group. This change was accompanied by DNA fragmentation that was an indicator of apoptosis (Fig. 4B).

**Figure 5:**
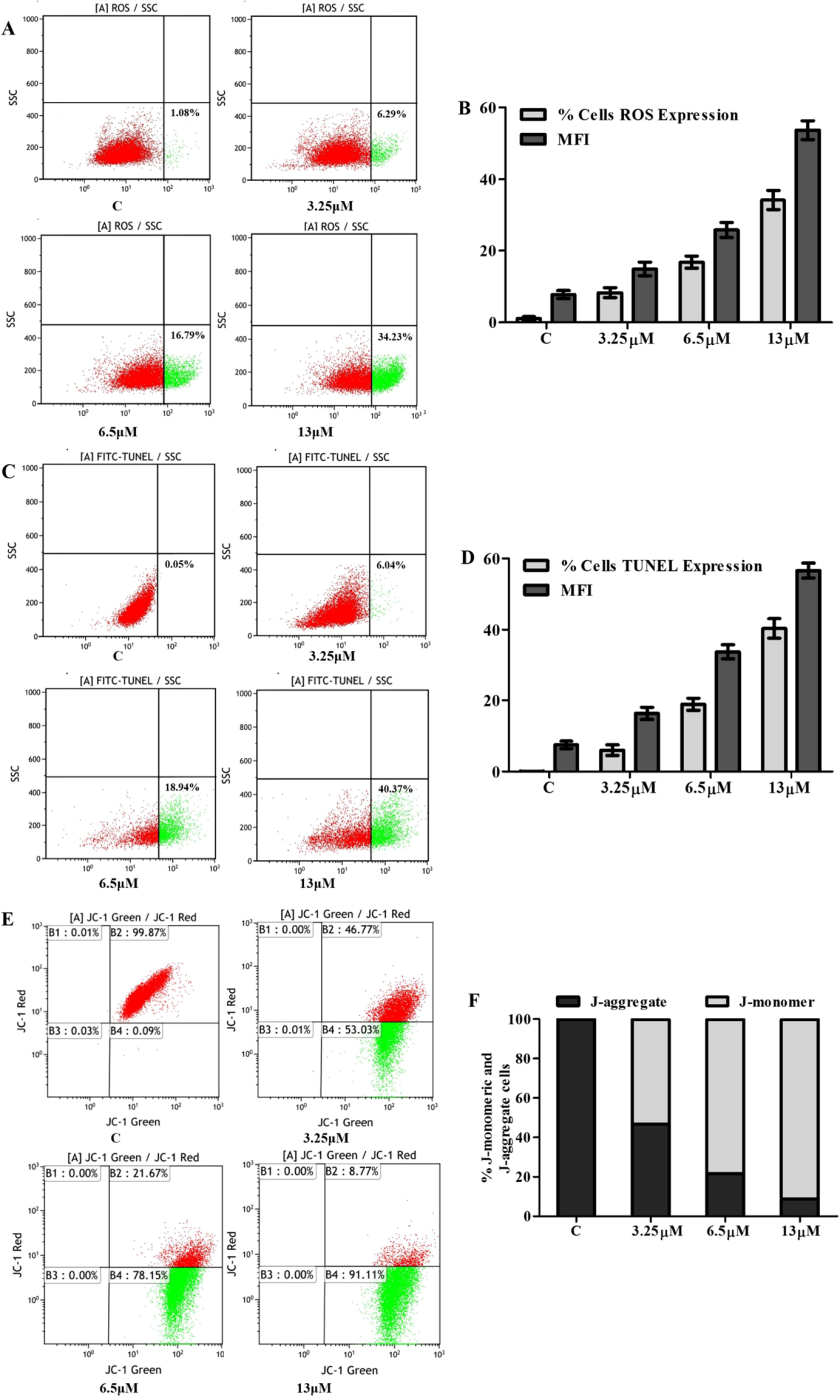
Effect of M22 on intracellular ROS levels, DNA fragmentation and mitochondrial transmembrane permeability (MMP, ΔΨm) changes. A549 cells incubated with M22 at different concentrations for 48 h were stained with DCFH-DA, fluorescein isothiocyanate-dUTP and JC-1 dye. The signal were assessed from fluorescence by flow cytometry and determined in terms of mean fluorescence intensity (MFI). Dot plots show that (**A**) ROS and (**C**) DNA fragmentation levels increases in a concentration-dependent manner. Bar diagram show that (**B**) percentage of elevated ROS cells, ROS production and (**D**) percentage of cells with TUNEL expression, DNA fragmentation degree increases in a dose-dependent manner. (**E**) Dot plots show that ΔΨm decreases in a dose-dependent manner. (**F**) Bar diagram showing percentage of apoptotic and nonapoptotic cells are hown on the right side of each panel.

M22 also increased reactive oxygen species (ROS) in a dose dependent manner compared with vehicle-only treated cells (Fig. 5A,B). This was accompanied by a significant loss of mitochondrial membrane potential (MMP) (Fig. 5E,F). These changes were accompanied by DNA degradation that was a major indicator of apoptosis (Fig. 5C,D).

### Effect of M22 on genes and proteins involved in apoptosis

**Figure 6:**
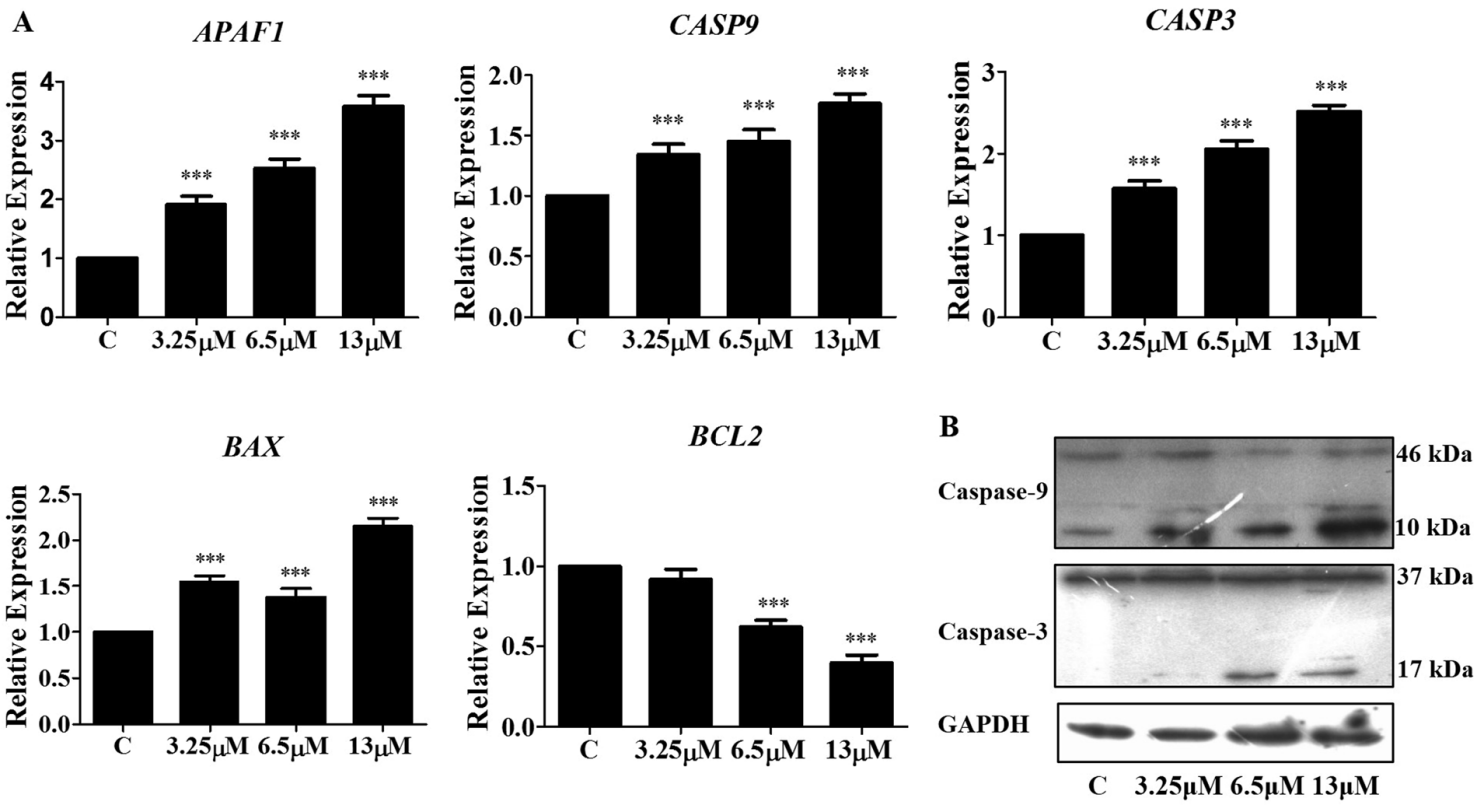
Effect of M22 on apoptosis related genes and proteins in A549 cells. (**A**) M22 upregulated the expression of proapoptotic genes *APAF1*, *CASP9*, *CASP3*, *BAX*, and depress antiapoptotic genes *BCL2* for 48 h. RNA was isolated and analysis was performed to detect by qRT-PCR. *Means P < 0.01, ***means P < 0.0001. (**B**) Western blot analysis showed cleavage of caspases-9 and −3 in M19 treated A549 cells after 48 h of exposure. Equal loading was confirmed by reprobing the membrane with GAPDH. The bands shown here are from a representative experiment repeated three times with similar results.

Expression of genes involved in the control of programmed cell death was evaluated in A549 cells after exposure to M22 for 48 h. We measured the steady state mRNA levels of *BCL*-2, *BAX*, *APAF*1, *CASP*9 and *CASP*3. All four of the apoptotic genes were upregulated while expression of the anti-apoptotic gene *BCL*-2 was lowered (Fig. 6A). In support of this, M22 increased cleavage of pro-Caspases 9 (46 kDa) and 3 (37 kDa) to their active forms. The cleaved caspases 9 (10 kDa) and 3 (17 kDa) are the characteristic hallmarks of apoptosis, which were detected in this study (Fig. 6B). Together these data indicated that M22 induces apoptosis.

## Discussion

Lupeol treatment of cells previously showed a certain degree of cytotoxic, anti-inflammatory and tumor pro-apoptotic activities^14,15^. Lupeol could induce apoptosis in A431 and LNCaP cells through the mitochondrial cell death pathway^16,17^. The derivative M22 was reported to be about 5 times more active^12^. In the present study, we explored the underlying anti-tumor mechanism of M22 on two principal aspects, cell cycle, apoptosis, related genes and proteins.

We found that M22 lowered *CDC25A* mRNA and protein levels were consistent with a G1 block. The CDC25A protein prevents dephosphorylation of Cdk2 and leads to the inactivation of the Cdk2/Cyclin E complex that triggers the G1 to S transition^18^. A decrease in CDC25A is consistent with our findings of a G1 block. Cdk2 activity was further regulated by p21^waf1/cip1^ although we found no significant changes in the mRNA levels of p21^waf1/cip1^. However, cyclin D1 mRNA and protein levels were decreased by M22. The release of the p21^waf1/cip1^ CDK2 inhibitor from the disrupted cyclin D1/Cdk4/6 complexes would result in the inactivation of the S-phase-promoting cyclin E /CDK2^19,20^ complex. This was also consistent with our results with a G1 block as the result of inactivation of the S-phase promoting complex. Besides, M22 treatment reduced expression of PCNA genes that would interfere with DNA replication and repair resulting in a G1 /S arrest^21–23^.

We also found an increase in *BAX* mRNA expression with a concomitant decrease in *BCL*2 expression following M22 treatment, indicative of apoptosis. M22 significantly increased ROS as MMP gradually decreased. This would result in cytochrome c release from mitochondria leading to decreased levels of ATP^24^. Cytochrome c binding to Apaf-1 would activate Caspase 9^25–27^ in the presence of sufficient ATP or dATP. Caspase 9 was involved in the activation cascade responsible for the execution of apoptosis and Caspase 9 binding to Apaf-1 enables the latter to cleave and activate Caspase 3. Caspase 3 activation was critical for apoptosis in A549 cells because this led to nuclear DNA fragmentation. Here we showed that up-modulation of Caspases 9 and 3, Apaf1 and Bax with the down-regulation of Bcl-2. This may represent a novel pathway through which M22 induces apoptosis.

## Conclusions

**Figure 7:**
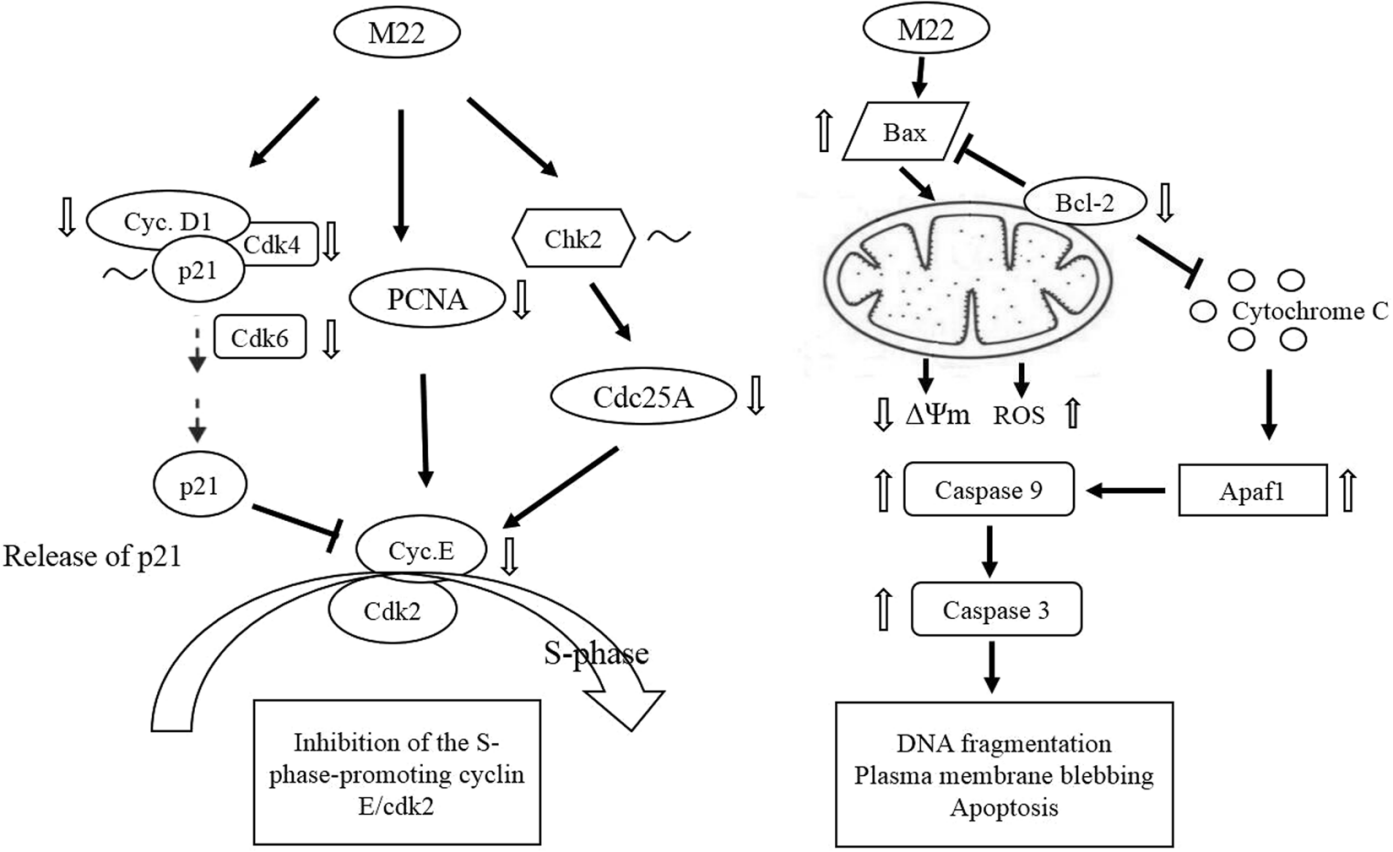
Proposed mechanism of action of M22 in the cell growth regulation of A549 cells. ΔΨm, change in mitochondrial transmembrane potential; ↑, upregulated expression; ↓, downregulated expression; ~, no change in expression.

M22 treatment of cell cultures showed significant cytotoxicity, increased the population of sub-G1 cells and attenuated the expression of Bcl-2, Bax, pro-Caspase 9 and pro-Caspase 3. The drug also suppressed the mRNA expression of G1 arrest-related genes of cyclin D1, CDC25A and PCNA and significantly changed the expression of ROS and MMP levels in A549 cells (Fig. 7).

## Methods

### Reagents and Cells

M22 was synthesized in our own laboratory with a purity of 99% as assessed by HPLC. M22 (C_34_H_58_N_2_O, MW 510.84) with a purity over 98% by HPLC was dissolved in DMSO and stored as small aliquots at −80 °C and then diluted in cell culture medium as needed. The human lung cancer cell line A549 was obtained from the Kunming Institute of Zoology, Chinese Academy of Sciences. Cells were maintained in RPMI1640 medium supplemented with 10% fetal bovine serum, streptomycin and penicillin (Gibco, Grand Island, NY, USA).

### Cytotoxicity

M22 cytotoxic effects were evaluated using a commercial MTT assay using the protocol supplied by the manufacturer (Sigma Chemical Co., St. Louis, MO, USA). Cells were seeded in 96-well microplates at a density of 1 × 10^4^ cells/well and treated with M22 for 48 h or 72 h at 2.5, 5, 10, 20, 40 and 80 μM. In addition, cells were also treated with various concentrations (2.5, 5, 10, 20, 40 and 80 μM) of lupeol and doxorubicin as the positive control for 48 h, respectively. Inhibitory ratio (%) = [OD (Control) − OD (Sample)]/ [OD (Control) − OD (Blank)] × 100. The anticancer activity of each sample was calculated and expressed as the concentration of compound that achieved 50% inhibition (IC_50_) to cancer cells.

### Colony formation assay

The A549 cells were treated with different concentrations of M22. Then the cells were incubated for an additional 10 days. Treatments were carried out in triplicate. The colonies obtained were 4% paraformaldehyde (Leagene, Beijing, China) fixed and stained with crystal violet (Leagene, Beijing, China). The colonies were counted and compared with untreated cells.

### Cell cycle and apoptosis

Cells were fixed in 75% ethanol and incubated with 0.1% RNaseA in PBS at 37 °C for 30 min, then suspended in PBS containing 50 μg/ml propidium iodide (PI) for 30 min at room temperature. The stained cells were analyzed for DNA content by flow cytometry (FACS Calibur, Becton Dickinson, Franklin Lakes, NJ, USA) using the Cell Quest software program supplied with the instrument.

Cell apoptosis was determined by flow cytometry using annexin-V/PI staining (Biotine, Shanghai, China). Briefly, A549 cells were seeded in 6-well plates with density of 3 × 10^5^ cells/well for 24 h and treated with M22 for 48 h. The cells were harvested and labeled with annexin V - fluorescein isothiocyanate solution and PI in binding buffer^28^. Cell fluorescence intensity was measured by flow cytometry as above. The analysis was repeated three times and the apoptosis rate was (%) for each M22 treatment was obtained.

### Protein and mRNA Expression

Western blot analysis used cells lysed in RIPA buffer (Biotine, Shanghai, China) and total protein was quantified using a BCA assay (Biomed, Beijing, China). Proteins were separated in an 8–10% denaturing polyacrylamide gradient gel. Proteins were transferred to PVDF membranes (Millipore, Bedford, MA, USA)^29^. The membrane was probed with antibodies for Cyclin D1, CDC25A, Caspase 3, Caspase 9 and GAPDH (Affinity Biosciences, Cincinnati, OH, USA) followed by exposure to horseradish peroxidase-conjugated anti-mouse or anti-rabbit antibodies as appropriate (Earth Ox, San Francisco, CA, USA). Protein expression was determined using an Enhanced Chemiluminescence kit (Bio-Rad, Hercules, CA, USA). Total RNA was isolated using an RNeasy mini kit (Qiagen, Valencia, CA) according to the manufacturer’s instructions. RNA was reverse transcribed using TransScript One-Step gDNA Removal and cDNA Synthesis SuperMix (TransGen Biotech, Beijing, China) and qPCR was performed using primers listed in Table 2 with the BAX System Q7 instrument (DuPont Nutrition & Health, Wilmington, DE, USA) according to the manufacturer’s protocol. The mRNA level of GAPDH was used to normalize the expression of genes of interest.

**Table 2:** Primers used for qPCR.

Target gene	Sequence (5′ to 3′)
*CCND1*	GCCCAGCAGAACATGGACC, GTGGGTGTGCAAGCCAGGT
*CCNE1*	GCCATGTTGTCTGAACAAAATAGG, TGCTCTGCTTCTTACCGCTC
*CDK4*	GGCCTCGAGATGTATCCCTG, CATCCTTATGTAGATAAGAGTGC
*CDK6*	AGCCAAAAGAATATCTGCCTACA, GGCTGTATTCAGCTCCGAGGT
*CDC25A*	GGGGATACAAGGAGTTCTTTATGAA, TTCAGACGACTGTACATCTCCCTCT
*PCNA*	AAGCCACTCCACTCTCTTCAACG, CCAAGTAGTATTTTAAGTGTCCCA
*APAF1*	ATGGACACCTTCTTGGACGACAG, TGTGGGGGCGGACAACTAA
*GAPDH*	TGGGCTACACTGAGCACCAG, TCCACCACCCTGTTGCTGTAG
*BCL2*	TCGCCCTGTGGATGACTGA, CAGAGACAGCCAGGAGAAATCA
*BAX*	TGCTTCAGGGTTTCATCCAG, GGCGGCAATCATCCTCTG
*P53*	CCCAGCCAAAGAAGAAACCA, AGCTGAATGAGGCCTTGGAA
*CASP3*	ACATGGCGTGTCATAAAATACC, CACAAAGCGACTGGATGAAC
*CASP9*	CCAGAGATTCGCAAACCAGAGG, GAGCACCGACATCACCAAATCC

### ROS and MMP and TUNEL assays

Briefly, A549 cells were suspended in PBS supplemented with 50 mM glucose for ROS detection and incubated with 10 mM DCFH-DA (Biotine, Shanghai, China) at 37 °C for 1 h^30^. The signal was assessed from fluorescence by flow cytometry (FACS Calibur, Becton Dickinson, Franklin Lakes, NJ, USA) and determined in terms of mean fluorescence intensity (MFI). The mitochondrial membrane potential was analyzed by incubating A549 cells with 5, 5′,6,6′-tetrachloro-1,1′,3,3′ tetraethylbenzimidazolylcarbocya-nine iodide/chloride (JC-1), a cationic dye that exhibits potential-dependent accumulation in mitochondria, indicated by fluorescence emission shift from red (590 nm) to green (525 nm) (Biotine, Shanghai, China). After M22 treatment, cells were stained with JC-1 and analyzed by a flow cytometry (FACS Calibur, Becton Dickinson, Franklin Lakes, NJ, USA). The ratio of mean fluorescence intensity (MFI) of red to green fluorescence was calculated for each treatment and plotted^31^.Values were given in terms of mean fluorescence intensity (MFI). DNA degradation was measured using a terminal deoxynucleotidyl transferase isothiocyanate-dUTP nick-end labeling (TUNEL) (TransGen Biotech, Beijing, China). Single cell suspension of treated and untreated A549 cells was washed with PBS and the cells (1 × 10^6^) were fixed overnight in ethanol (90%). The cells were washed and labeled with fluorescein-dUTP for 1h^32^. The signal were assessed from fluorescence by flow cytometry (FACS Calibur, Becton Dickinson, Franklin Lakes, NJ, USA) and determined in terms of mean fluorescence intensity (MFI).

### Statistical analyses

Data were presented as means ± standard deviation (SD) of three or more replicates. Statistical significance was verified by Dunnett’s multiple comparison tests using GraphPad Prism 5 software (GraphPad, San Diego, CA, USA).

## Electronic supplementary material

Supplementary information
