# Supplementary material for: Regulation of Signaling Pathways Involved in the Anti-proliferative and Apoptosis-inducing Effects of M22 against Non-small Cell Lung Adenocarcinoma A549 Cells
Source: Sci Rep. 2018 Jan 17;8:992. doi: 10.1038/s41598-018-19368-0 (PMC5772365; doi:10.1038/s41598-018-19368-0)
Supplement: Supplementary file 1 — Supplementary information [file 41598_2018_19368_MOESM1_ESM.doc]

**Supplementary information**

Regulation of signaling pathways involved in anti-proliferative and apoptosis-inducing effect of M22 against non-small cell lung

adenocarcinoma A549 Cells

Yao Yuan^a,b#^, Jiewei Wu^a,b#^, Bailing Li^a,b^, Jia Niu^a,b^, Haibo Tan^a^,*, Shengxiang Qiu^a^,*

*^a^ Key Laboratory of Plant Resources Conservation and Sustainable Utilization, Guangdong Provincial Key Laboratory of Applied Botany, South China Botanical Garden, Chinese Academy of Sciences, Guangzhou 510650, (P. R.China)*

*^b^ University of Chinese Academy of Sciences, Beijing, 100049 (P. R.China)*

^#^ Both authors contributes equally to this paper.

* Corresponding author

Haibo Tan, PhD, Assistant researcher professor; Shengxiang Qiu, PhD, Professor

Tel.: +86 20 3708119

E-mail: [haibotan09@163.com](mailto:haibotan09@163.com); shengxiangqiu@163.com

**Supplemented information**

^1^H NMR Data

Table 1**.** The list of primers used in qPCR.

Figure. 1. Chemical structure and cytotoxicity of M22. (A) Chemical structure of compound M22. (B–C) Dose- and time-dependent effect of M22 on inhibition of A549 cells, analyzed by MTT assay. (D) A549 and MRC5 cells were cultured with various concentrations of M22 for 48h and cell inhibition was analyzed by MTT assay. The experiment was repeated three times and the data are presented as mean ± S.D. (E) Colony forming ability of A549 cells was inhibited by M22 (3.25μM, 6.5μM and 13 μM) treatment for 10 days.

Figure 2. Flow cytometry analysis histograms of G0/G1-phase arrest in 24 h M22 treated A549 cells. (A) The G0/G1 peak increases with corresponding decrease in S and G2 peak in dose dependent manner. M22 treated cells at 13μM showing an increased sub G1 peak indicating apoptosis. (B) The percent of cells in different phases of cell cycle are represented by bar diagram. Data are from 3 independent experiments and analyzed by one-way analysis of variance, *** p < 0.001 compared to control. PI ﬂuorescence was measured using a ﬂow cytometer with FL-2 ﬁlter.

Figure 3. M22 regulates G0/G1 arrest through a negative feedback mechanism. (A) M22 attenuates mRNA expression of Cyclin D1, Cyclin E1, CDK4, CDK6, CDC25A and PCNA genes in A549 cells for 24 h. RNA was isolated and analsis was performed to detect by qRT-PCR. * means P <0.01, *** means P <0.0001. (B) M22 down-regulates the expression of Cyclin D1 and CDC25C protein in A549 cells by Western blotting.

Figure 4. Detection of apoptosis induced by M22 by flow cytometry and confocal microscopy. (A) Evaluation of apoptosis by Annexin V/PI dual staining assay. Numbers of Annexin V-and PI-positive cells were determined using ﬂow cytometry. Quadrant 1: necrotic cells; Quadrant 2: late-apoptotic cells; Quadrant 3: viable cells; Quadrant 4: early apoptotic cells. (B) The percent of apoptotic cells are represented by bar diagram. (C) Morphologic changes of A549 cells treated by M22 for 48h were fixed and stained with Hoechst 33258. M22 treated cells show apoptotic, condensed and fragmented fluorescent nuclei. Bars, 10 μm.

Figure 5. Effect of M22 on intracellular ROS levels, DNA fragmentation and mitochondrial transmembrane permeability (MMP, ΔΨm) changes. A549 cells incubated with M22 at different concentrations for 48h were stained with DCFH-DA, fluorescein isothiocyanate-dUTP and JC-1 dye. The signal were assessed from fluorescence by flow cytometry and determined in terms of mean ﬂuorescence intensity (MFI). Dot plots show that (A) ROS and (C) DNA fragmentation levels increases in a concentration-dependent manner. Bar diagram show that (B) percentage of elevated ROS cells, ROS production and (D) percentage of cells with TUNEL expression, DNA fragmentation degree increases in a dose-dependent manner. (E) Dot plots show that ΔΨm decreases in a dose-dependent manner. (F) Bar diagram showing percentage of apoptotic and nonapoptotic cells are hown on the right side of each panel.

Figure 6. Effect of M22 on apoptosis related genes and proteins in A549 cells. (A) M22 upregulated the expression of proapoptotic genes *APAF1*, *CASP9*, *CASP3*, *BAX*, and depress antiapoptotic genes *BCL2* for 48h. RNA was isolated and analsis was performed to detect by qRT-PCR. * means P <0.01, *** means P <0.0001. (B) Western blot analysis showed cleavage of caspases-9 and -3 in M19 treated A549 cells after 48 h of exposure. Equal loading was conﬁrmed by reprobing the membrane with GAPDH. The bands shown here are from a representative experiment repeated three times with similar results.

Figure 7. Proposed mechanism of action of M22 in the cell growth regulation of A549 cells. ΔΨm, change in mitochondrial transmembrane potential; ↑, upregulated expression; ↓, downregulated expression; ~, no change in expression.

^1^H NMR Data

1H-NMR (CDCl_3_, 300 MHz) *δ* 4.89, 4.87 (each 1H, dd, J = 2.4, 1.5 Hz), 3.20 (1H, dd, J = 10.8, 5.2), 2.92 (1H, d, J = 14.3 Hz), 2.83 (1H, d, J = 14.3 Hz), 2.31 (3H, s), 1.75-1.25 (m), 2.02 (1H, m), 1.04, 0.98, 0.95 (each 3H, s), 0.84, 0.79, 0.77 (each 3H, s).

**Table 1. IC_50_ values of lupeol derivatives M22 against four human cancer cell lines for 48h.**

| **Sample** | **IC_50_ (μM)** | | | |
| --- | --- | --- | --- | --- |
|  | **A549** | **SW480** | **HepG2** | **HeLa** |
| M22 | 6.80 ± 0.33 | 9.74 ± 0.43 | 9.64 ± 0.25 | 7.18 ± 0.36 |
| Lupeol | 35.69 ± 0.75 | 48.51 ± 0.82 | 45.23 ± 0.67 | 34.76 ± 0. 64 |
| Doxorubicin | 25.43 ± 0.57 | >50 | >50 | 25.23 ± 0. 53 |

Table 2**.** The list of primers used in qPCR.

| **Target gene** | **Sequence of primer** |
| --- | --- |
| *CCND1* | forward:5`-GCCCAGCAGAACATGGACC-3`  reverse: 5`-GTGGGTGTGCAAGCCAGGT-3` |
| *CCNE1* | forward:5`-GCCATGTTGTCTGAACAAAATAGG-3`  reverse: 5`-TGCTCTGCTTCTTACCGCTC-3` |
| *CDK4* | forward:5`-GGCCTCGAGATGTATCCCTG-3`  reverse: 5`-CATCCTTATGTAGATAAGAGTGC-3` |
| *CDK6* | forward:5`-AGCCAAAAGAATATCTGCCTACA-3`  reverse: 5`-GGCTGTATTCAGCTCCGAGGT-3` |
| *CDC25A* | forward:5`-GGGGATACAAGGAGTTCTTTATGAA-3`  reverse: 5`-TTCAGACGACTGTACATCTCCCTCT-3` |
| *PCNA* | forward:5`-AAGCCACTCCACTCTCTTCAACG-3`  reverse: 5`-CCAAGTAGTATTTTAAGTGTCCCA-3` |
| *APAF1* | forward: 5`-ATGGACACCTTCTTGGACGACAG-3`  reverse: 5`-TGTGGGGGCGGACAACTAA-3` |
| *GAPDH* | forward:5`-TGGGCTACACTGAGCACCAG-3`  reverse:5`-TCCACCACCCTGTTGCTGTAG-3` |
| *BCL2* | forward:5`-TCGCCCTGTGGATGACTGA-3`  reverse: 5`-CAGAGACAGCCAGGAGAAATCA-3` |
| *BAX* | forward:5`-TGCTTCAGGGTTTCATCCAG-3`  reverse: 5`-GGCGGCAATCATCCTCTG-3` |
| *P53* | forward:5`-CCCAGCCAAAGAAGAAACCA-3`  reverse:5`-AGCTGAATGAGGCCTTGGAA-3` |
| *CASP3* | forward:5`-ACATGGCGTGTCATAAAATACC-3`  reverse: 5`-CACAAAGCGACTGGATGAAC-3` |
| *CASP9* | forward:5`-CCAGAGATTCGCAAACCAGAGG-3`  reverse: 5`-GAGCACCGACATCACCAAATCC-3` |


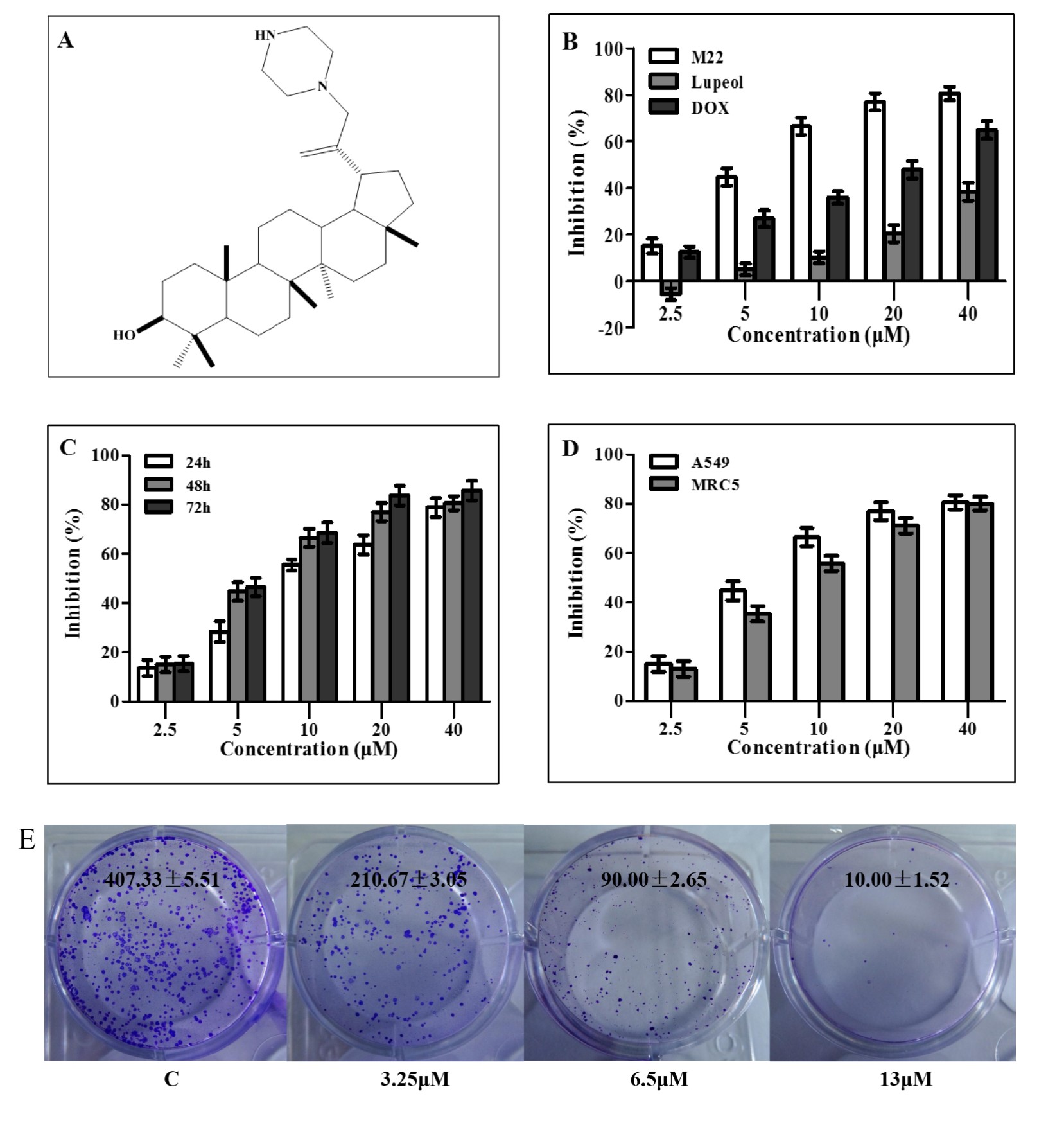


Figure. 1. Chemical structure and cytotoxicity of M22. (A) Chemical structure of compound M22. (B–C) Dose- and time-dependent effect of M22 on inhibition of A549 cells, analyzed by MTT assay. (D) A549 and MRC5 cells were cultured with various concentrations of M22 for 48h and cell inhibition was analyzed by MTT assay. The experiment was repeated three times and the data are presented as mean ± S.D. (E) Colony forming ability of A549 cells was inhibited by M22 (3.25μM, 6.5μM and 13 μM) treatment for 10 days.


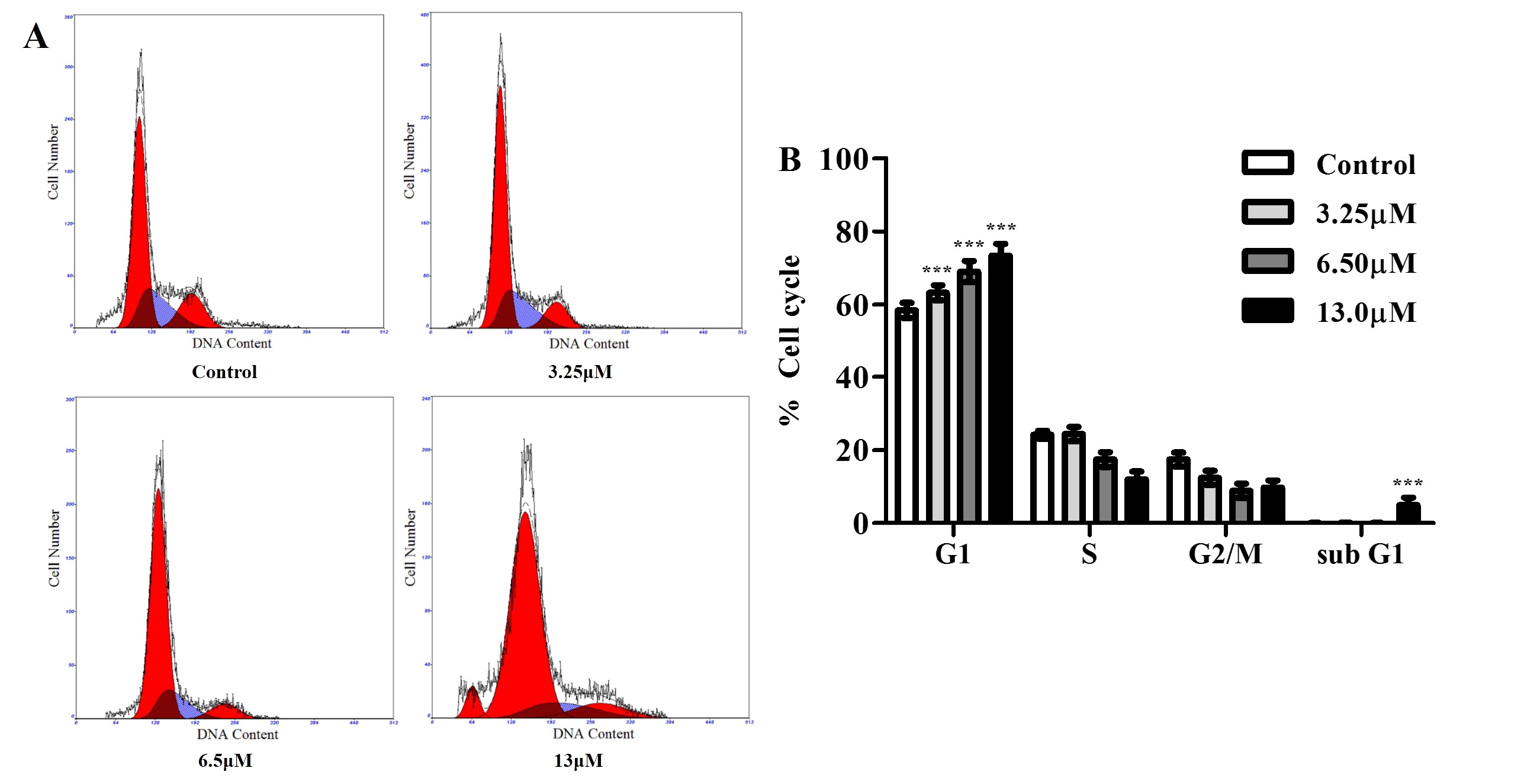


Figure 2. Flow cytometry analysis histograms of G0/G1-phase arrest in 24 h M22 treated A549 cells. (A) The G0/G1 peak increases with corresponding decrease in S and G2 peak in dose dependent manner. M22 treated cells at 13μM showing an increased sub G1 peak indicating apoptosis. (B) The percent of cells in different phases of cell cycle are represented by bar diagram. Data are from 3 independent experiments and analyzed by one-way analysis of variance, *** p < 0.001 compared to control. PI ﬂuorescence was measured using a ﬂow cytometer with FL-2 ﬁlter.


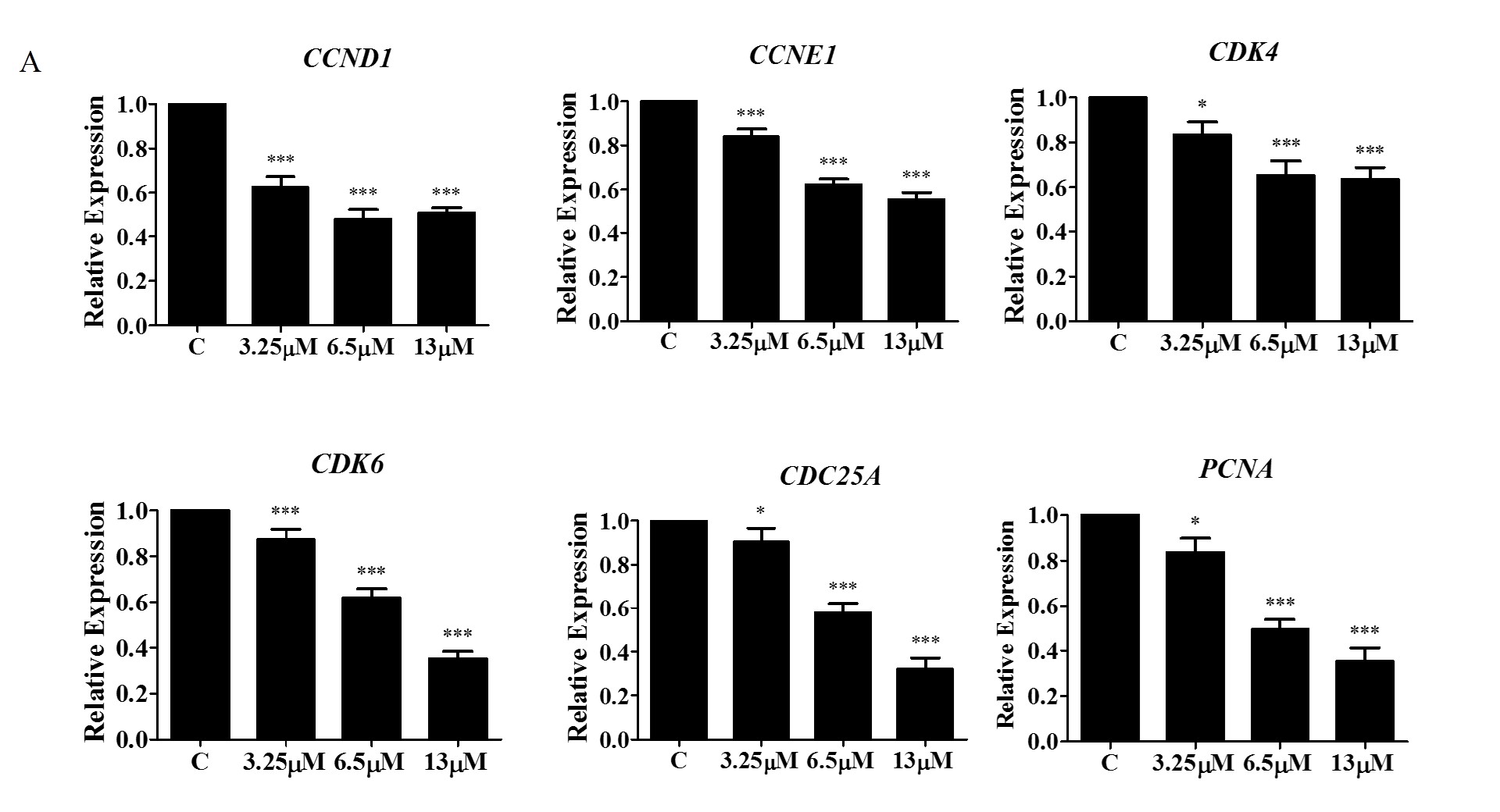


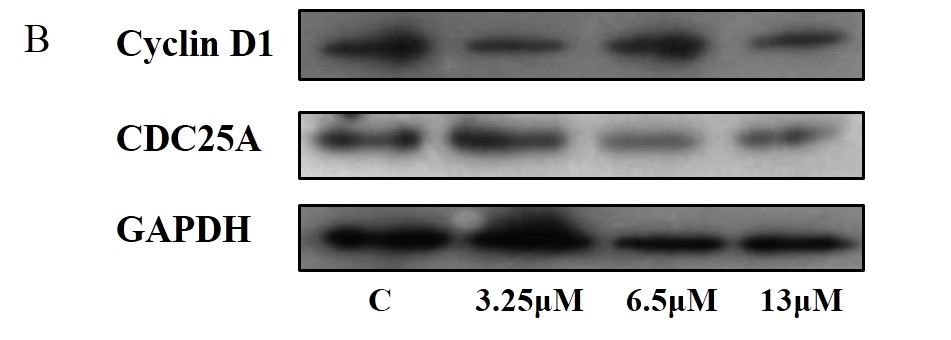


Figure 3. M22 regulates G0/G1 arrest through a negative feedback mechanism. (A) M22 attenuates mRNA expression of cyclin D1, cyclin E1, CDK4, CDK6, CDC25A and PCNA genes in A549 cells for 24 h. RNA was isolated and analysis was performed to detect by qRT-PCR. * means P <0.01, *** means P <0.0001. (B) M22 down-regulates the expression of cyclin D1 and CDC25A protein in A549 cells by western blotting.


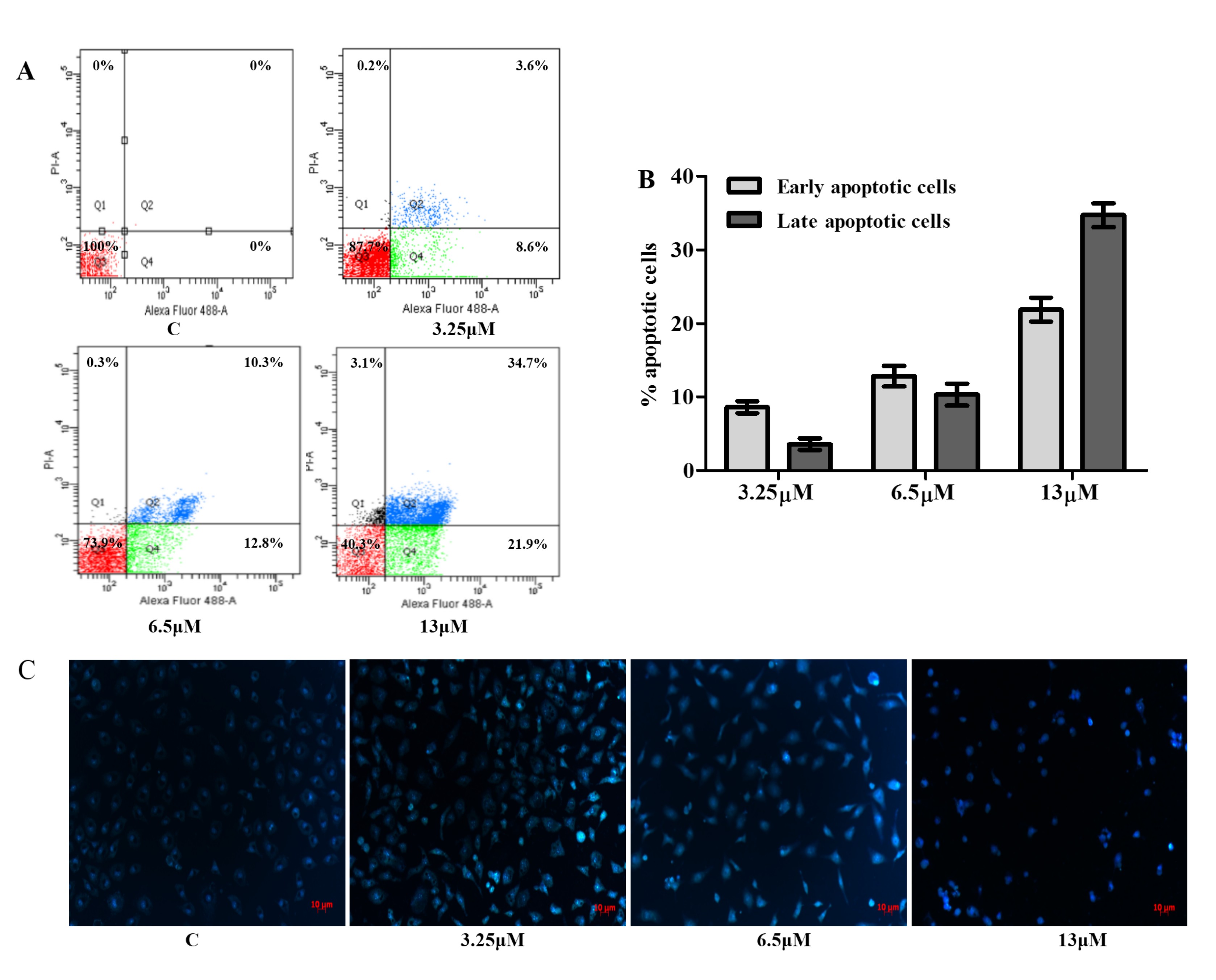


Figure 4. Detection of apoptosis induced by M22 by flow cytometry and confocal microscopy. (A) Evaluation of apoptosis by Annexin V/PI dual staining assay. Numbers of Annexin V and PI positive cells were determined using ﬂow cytometry. Quadrant 1: necrotic cells; Quadrant 2: late-apoptotic cells; Quadrant 3: viable cells; Quadrant 4: early apoptotic cells. (B) The percent of apoptotic cells are represented by bar diagram. (C) Morphologic changes of A549 cells treated by M22 for 48h were fixed and stained with Hoechst 33258. M22 treated cells show apoptotic, condensed and fragmented fluorescent nuclei. Bars, 10μm.


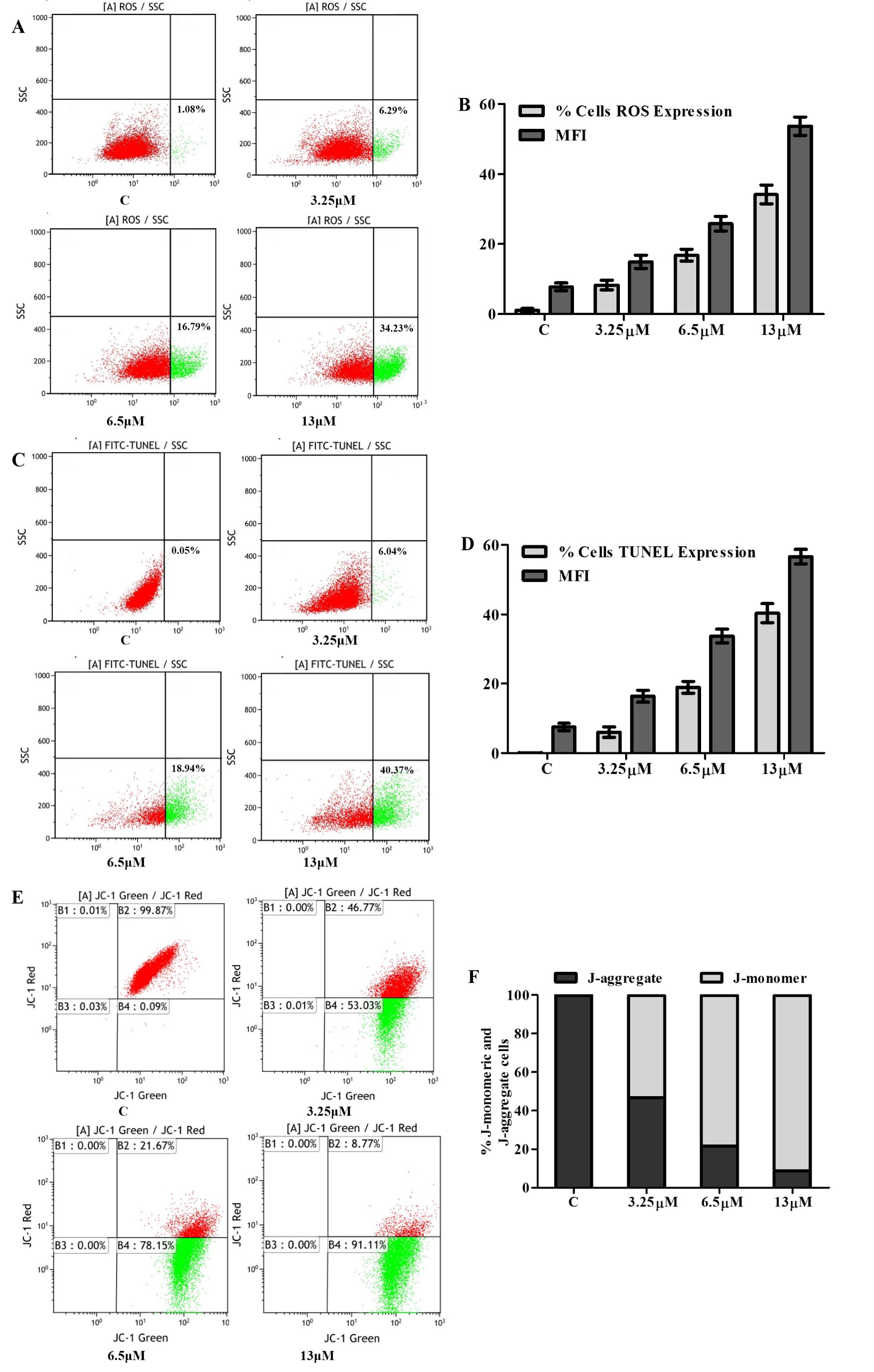


Figure 5. Effect of M22 on intracellular ROS levels, DNA fragmentation and mitochondrial transmembrane permeability (MMP, ΔΨm) changes. A549 cells incubated with M22 at different concentrations for 48h were stained with DCFH-DA, fluorescein isothiocyanate-dUTP and JC-1 dye. The signal were assessed from fluorescence by flow cytometry and determined in terms of mean ﬂuorescence intensity (MFI). Dot plots show that (A) ROS and (C) DNA fragmentation levels increases in a concentration-dependent manner. Bar diagram show that (B) percentage of elevated ROS cells, ROS production and (D) percentage of cells with TUNEL expression, DNA fragmentation degree increases in a dose-dependent manner. (E) Dot plots show that ΔΨm decreases in a dose-dependent manner. (F) Bar diagram showing percentage of apoptotic and nonapoptotic cells are hown on the right side of each panel.


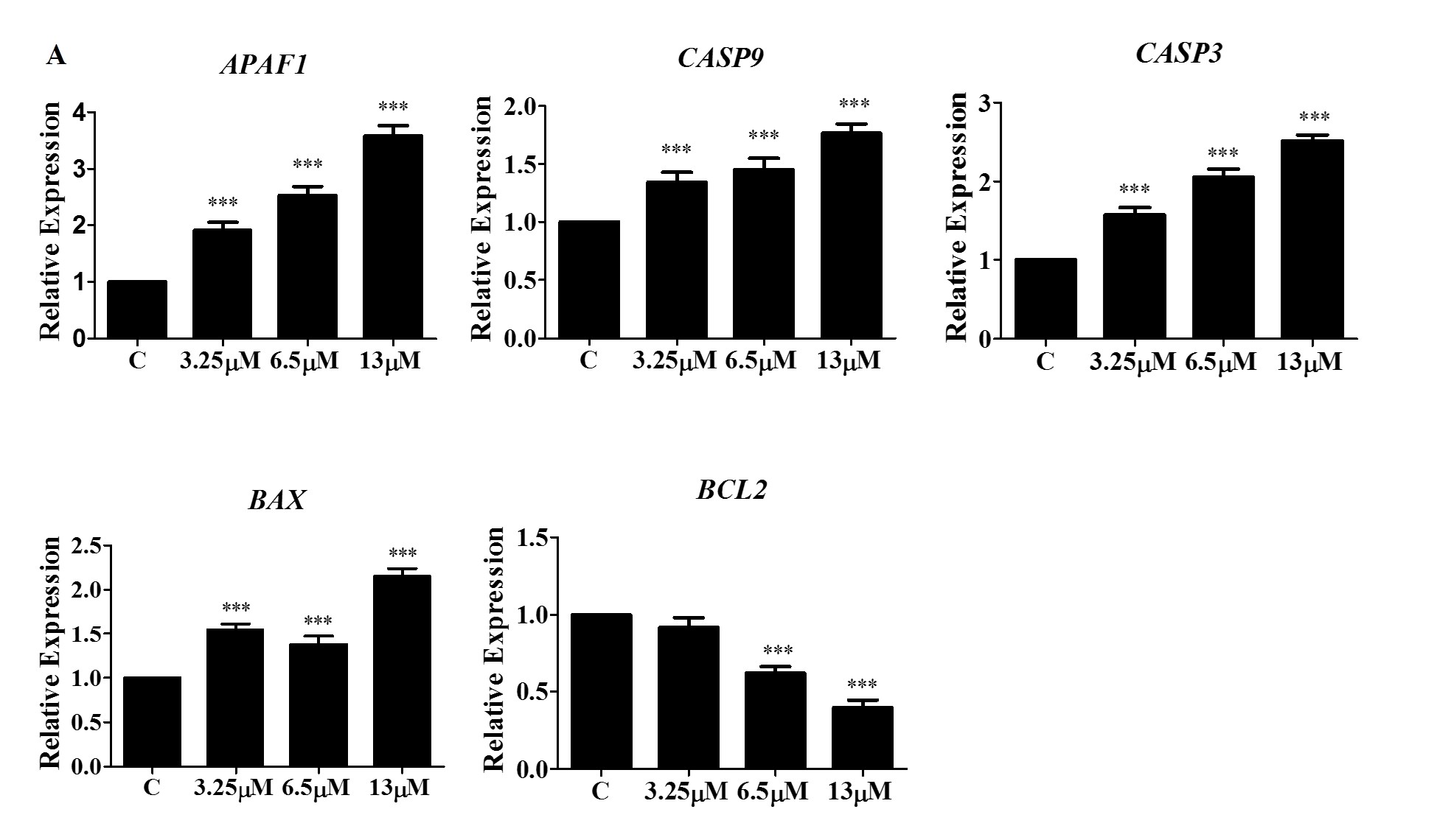


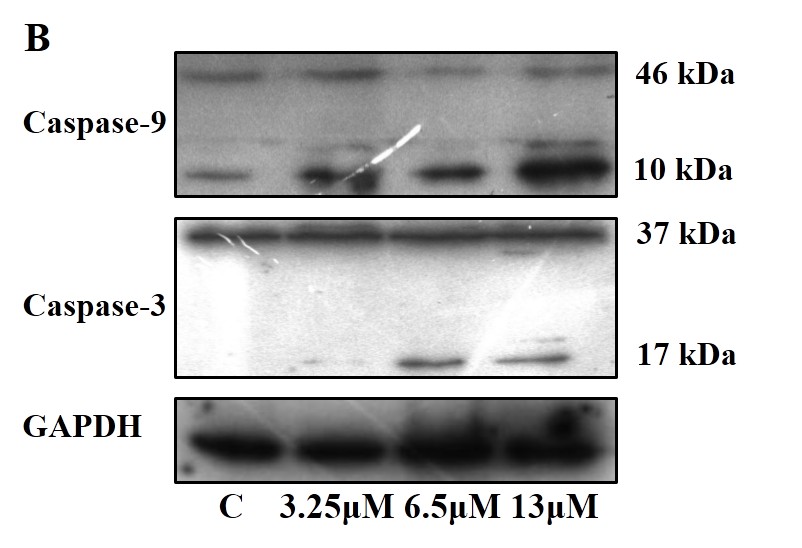


Figure 6. Effect of M22 on apoptosis related genes and proteins in A549 cells. (A) M22 upregulated the expression of proapoptotic genes *APAF1*, *CASP9*, *CASP3*, *BAX*, and depress antiapoptotic genes *BCL2* for 48h. RNA was isolated and analysis was performed to detect by qRT-PCR. * means P <0.01, *** means P <0.0001. (B) Western blot analysis showed cleavage of caspases-9 and -3 in M19 treated A549 cells after 48 h of exposure. Equal loading was conﬁrmed by reprobing the membrane with GAPDH. The bands shown here are from a representative experiment repeated three times with similar results.


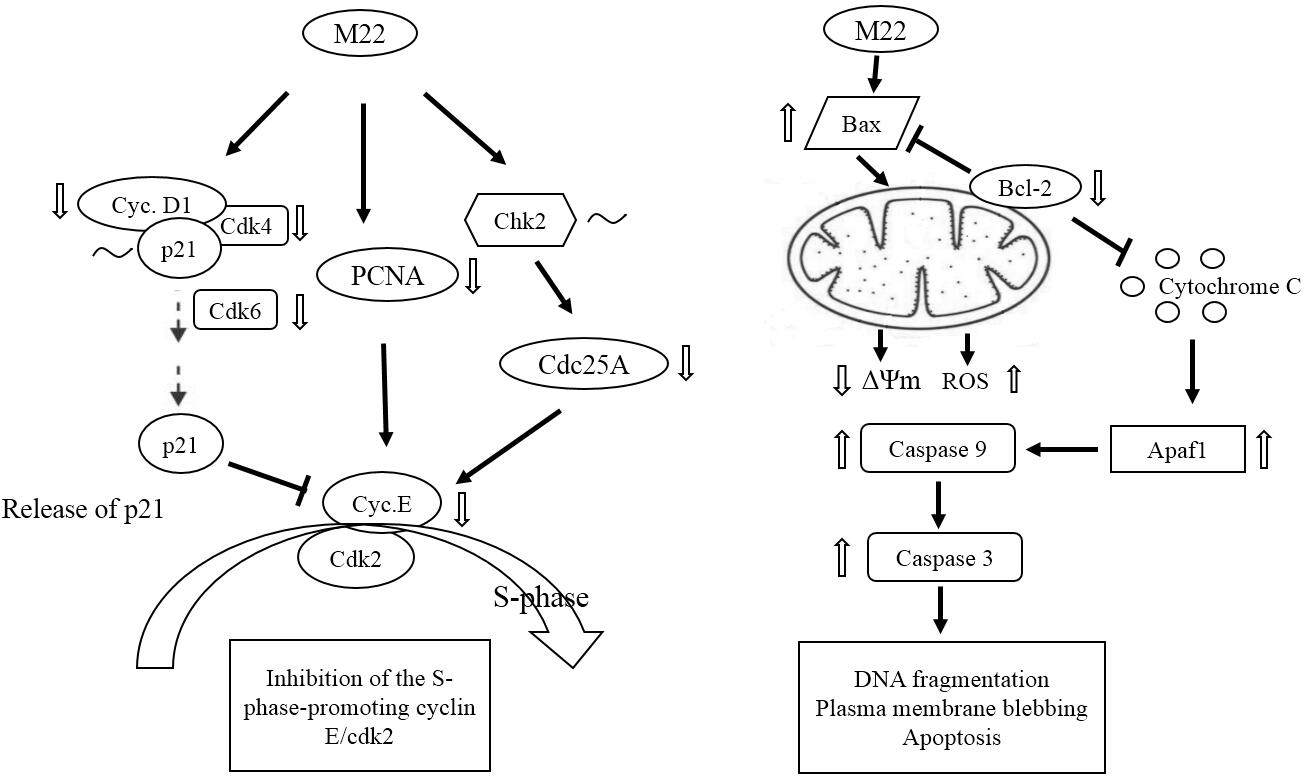


Figure 7. Proposed mechanism of action of M22 in the cell growth regulation of A549 cells. ΔΨm, change in mitochondrial transmembrane potential; ↑, upregulated expression; ↓, downregulated expression; ~, no change in expression.


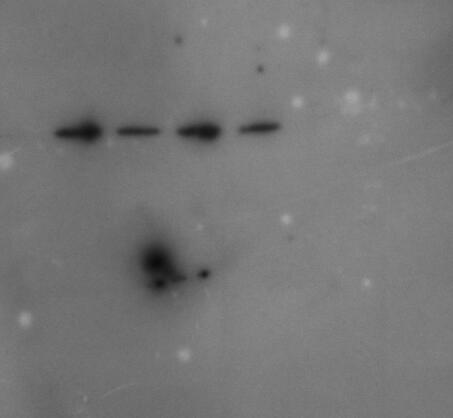


Figure 8 Original scans for the western blots of Cyclin D1 in Figure 3.


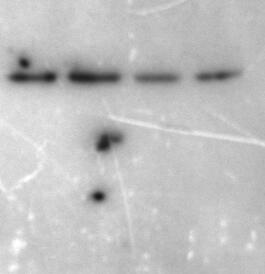


Figure 9 Original scans for the western blots of CDC25A in Figure 3.


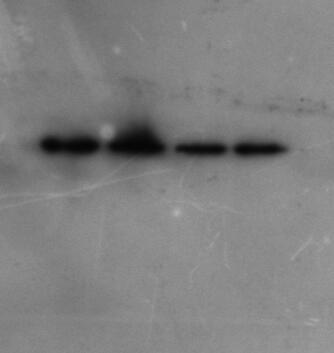


Figure 10 Original scans for the western blots of GAPDH in Figure 3.


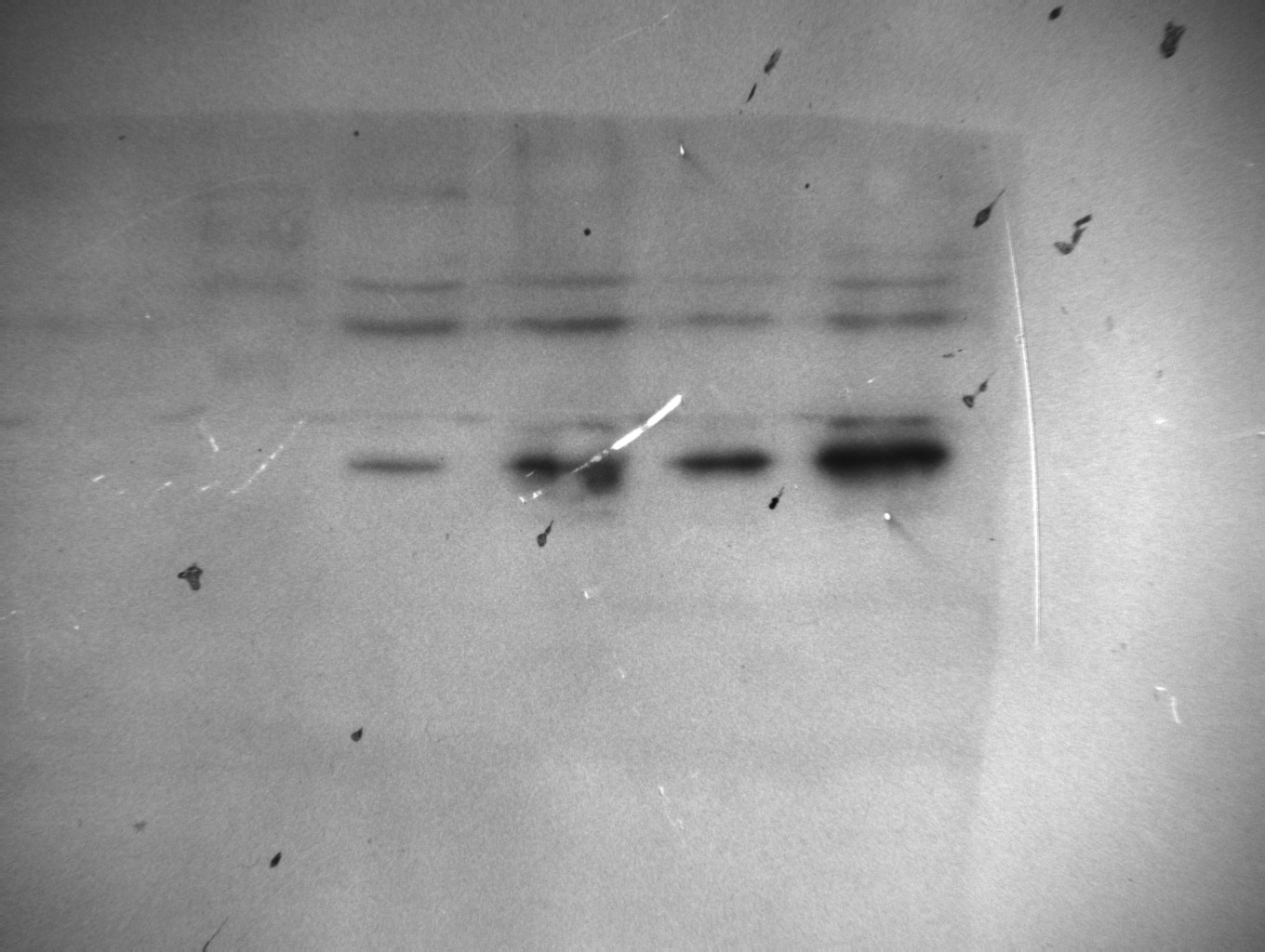


Figure 11 Original scans for the western blots of Caspase9 in Figure 6.


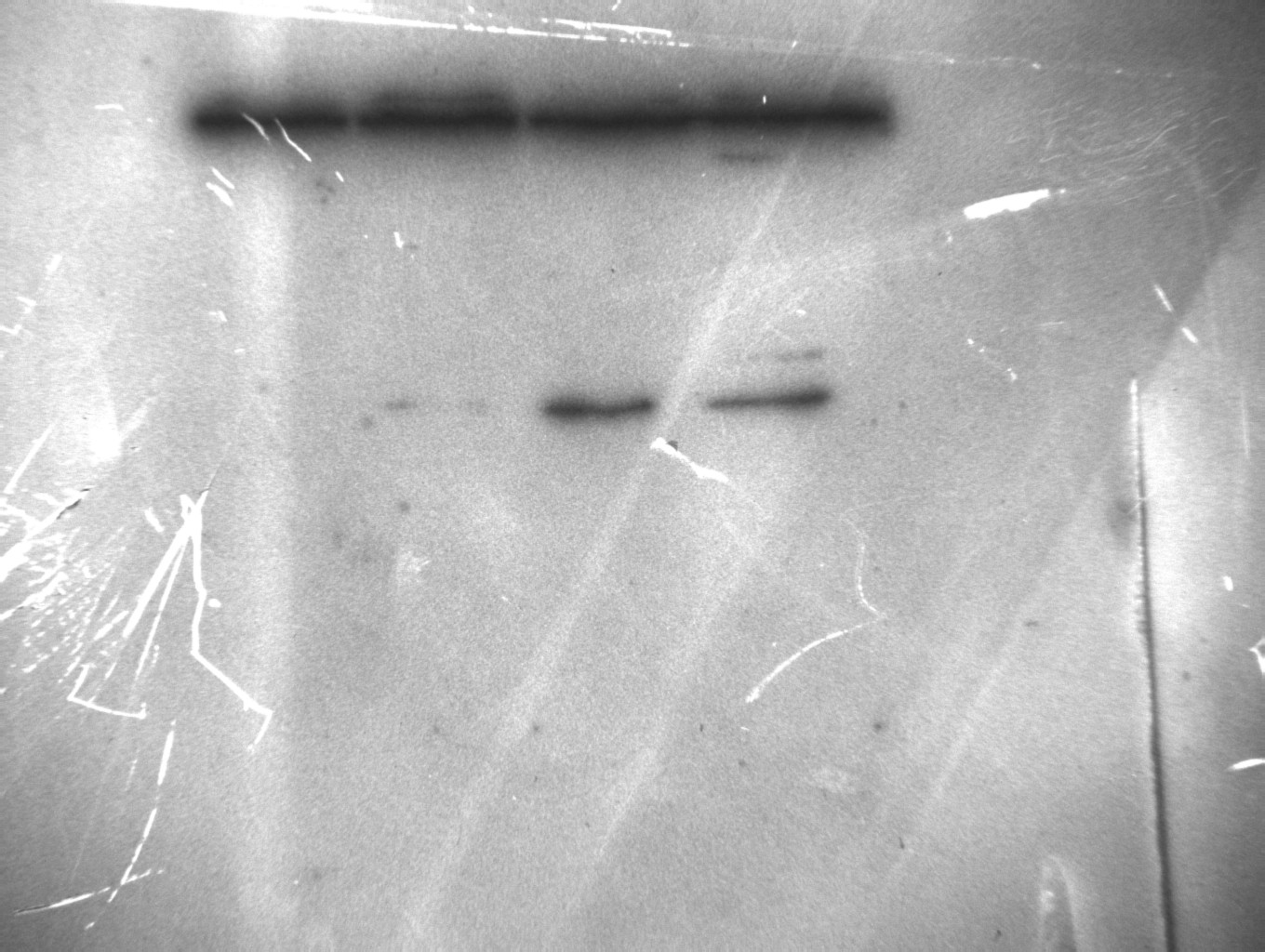


Figure 12 Original scans for the western blots of Caspase3 in Figure 6.


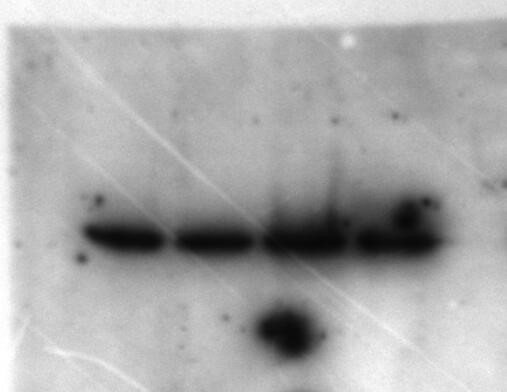


Figure 11 Original scans for the western blots of GAPDH in Figure 6.
